# Can We Use Routine Data for Strategic Decision Making? A Time Trend Comparison Between Survey and Routine Data in Mali

**DOI:** 10.9745/GHSP-D-21-00281

**Published:** 2021-12-31

**Authors:** Talata Sawadogo-Lewis, Youssouf Keita, Emily Wilson, Souleymane Sawadogo, Ibrahim Téréra, Hamadoun Sangho, Melinda Munos

**Affiliations:** a Institute for International Programs, Johns Hopkins Bloomberg School of Public Health, Baltimore, MD, USA.; b Institute for International Programs, Johns Hopkins Bloomberg School of Public Health, Bamako, Mali.; c Agence Nationale de Télésanté et d'Informatique Médicale, Bamako, Mali.; d Institut National de la Santé Publique (INSP), Bamako, Mali.

## Abstract

**Background:**

Countries with scarce resources need timely and high-quality data on coverage of health interventions to make strategic decisions about where to allocate investments in health. Household survey data are generally regarded as “gold standard,” high-quality data. This study assessed the comparability of intervention coverage time trends from routine and survey data at national and subnational levels in Mali.

**Methods:**

We compared 3 coverage indicators: contraceptive prevalence rate, institutional delivery, and 3 doses of diphtheria, pertussis, and tetanus (DPT3) vaccine, using 3 Mali Demographic and Health Surveys (DHS 2001, 2006, and 2012–2013) and routine health system data covering 2001–2012. For routine data, we used local health information system (HIS) annual reports and an HIS database. To compare time trends between the data sources, we calculated the percentage point change and 95% confidence interval from 2001–2006 and 2006–2012. We then computed the absolute and relative differences between the 2 data sources for each indicator over time at national and regional levels and assessed their level of significance.

**Results:**

The direction and magnitude of the time trends of contraceptive prevalence rate, institutional delivery, and DPT3 vaccine from 2001 to 2012 were similar at the national level between data sources. At the regional level, there were significant differences in the magnitude and direction of time trends for institutional delivery and the DPT3 vaccine; contraceptive prevalence trends were more consistent. Routine data tended to overestimate DPT3 coverage, and underestimate institutional delivery and contraceptive prevalence relative to survey data.

**Conclusion:**

Routine data in Mali—particularly at the national level—appear to be appropriate for use to inform program planning and prioritization, but routine time trends should be interpreted with caution at the subnational level. For program evaluations, routine data may not be appropriate to draw accurate inferences about program impact.

## Abstract (precis)

Routine data, which is available more regularly than the "gold standard" survey data, can be used to inform programmatic decisions in Mali at the national level. However, caution must be used if using data at a subnational level.

## Teaser

**Key Findings:**

- The direction and magnitude of the time trends of contraceptive prevalence rate, institutional delivery, and 3 doses of the diphtheria, pertussis, and tetanus (DPT3) vaccine from 2001 to 2012 were similar at the national level between data sources.
- At the regional level, routine data tended to overestimate DPT3 coverage, and underestimate institutional delivery and contraceptive prevalence relative to survey data.
- Routine data are appropriate for use for program planning. However, it does not seem to be appropriate for use for impact evaluations, particularly at the regional level.

**Key Implications:**

- Program planners and evaluators can continue to use routine data to detect overall trends but until the quality of routine data is improved, they should refrain from using it for impact evaluations.

Résumé en français à la fin de l'article.

## BACKGROUND

All countries, especially those with scarce resources, need to make strategic decisions about where to allocate investments in health. Timely, high-quality, population-based data on coverage of key health interventions are necessary to evaluate project or program impact and thus guide future efforts. Household survey data—including from U.S. Agency for International Development-funded Demographic and Health Surveys or United Nations Children's Fund-funded Multiple Indicator Cluster Surveys—are generally considered to use “gold standard” methods and, therefore, to produce high-quality population-based data.^1^^,^^2^ These data are publicly available and free to access. However, because the surveys are expensive to conduct, they are conducted relatively infrequently. Country decision makers must make frequent programmatic decisions and adjustments, and survey data, typically available every 3 to 5 years, cannot be used for this purpose. In addition, while survey data are typically available at the subnational level (e.g., regional level), they are usually not available for smaller geographic areas such as districts.^1^

To overcome these limitations, country-level planners often turn to routine data sources to fill these data gaps. Routine data collected by the health system are typically available on a monthly or quarterly basis. If properly collected and managed, they allow stakeholders to observe changes in coverage from year to year that can be important for time-sensitive decision making. At the district health facility level, they serve as an appropriate source for operational decision making. Because they are collected and managed by national staff from the health system, their collection is more sustainable. Finally, routine data are relatively inexpensive to collect.^2^

Health information systems (HIS), which rely on routine data, can have significant limitations. Although many countries publish annual reports based on HIS data,^3^ it can be difficult to access the data underlying the reports.^4^ Some indicators of interest are not collected or are not aggregated in the HIS; when indicators are available, the data are sometimes of poor quality.^1^^,^^5^^,^^6^ In many countries, the HIS is limited to data from public health facilities; data from private facilities are not included. For intervention coverage measures, the denominators—the estimates of the population in need —are often based on census projections. The accuracy of these projections can be affected by factors like the time since the last census and internal population movements.

Despite these well-documented limitations, routine data are often the de facto data source used for programmatic planning in low- and middle-income countries, particularly where no recent household survey exists. Within the context of the Global Affairs Canada-funded National Evaluation Platform—dedicated to improving evidence-based decision making^7-10^—a team of Malian researchers found that at least 5 major maternal, newborn, and child health and nutrition (MNCHN) programs rely on HIS data as their source of coverage indicator data in Mali.^11^ A 2013 evaluation of Mali's HIS concluded that it had poor data quality in general, due in large part to poor data archiving and uneven record keeping. Regional HIS data were also found to be of generally higher quality than district-level data.^12^ Reducing maternal, newborn, and under-5 mortality is a priority for the Government of Mali, and data are needed to inform this work. Mali's decennial plan for health and social development (*Plan Décennal de Développement Sanitaire et Social* 2014–2023) recognizes the need to increase routine data quality, timeliness, and use for decision making at all levels.^13^

Given that routine data are widely used for planning and evaluation and to fill the gaps in between household surveys in Mali, it is of interest to decision makers to understand the comparability of these data. While some differences in the levels of coverage indicators between the 2 data sources are expected (because routine data are limited to the public health sector and because of some differences in indicator definitions), it would be useful to know whether the HIS captures the same time trends as population-based surveys. To answer this question, we compared time trends in routine and household survey data from 2001 to 2012 in Mali for 3 indicators to inform the use of routine data by decision makers in Mali.

Because routine data are widely used for planning and evaluation and to fill the gaps in between household surveys in Mali, decision makers seek to understand the comparability of these data.

## METHODS

This analysis focused on 3 indicators: modern and traditional contraceptive prevalence rate (CPR), 3 doses of diphtheria, pertussis, and tetanus vaccine (DPT3), and institutional delivery. We focused on these because they were the most complete indicators across regions and years in the HIS and represented a range of services across the continuum of care.

### Data Sources and Quality Assessment

We used DHS data collected in 2001,^14^ 2006,^15^ and 2012–2013^16^ in Mali. Data were collected in all regions and the district of Bamako in 2001 and 2006. In 2012, the regions of Tombouctou, Gao, and Kidal, and 3 districts in the Mopti region (Douentza, Ténenkou, and Youwarou) were excluded due to security concerns.

For routine data, coverage estimates were obtained from HIS-validated annual reports. Numerators and denominators were double extracted in a standardized format for each indicator, year, region, and at the national level from 2001 to 2012. For each indicator, the numerator as reported in the HIS (e.g., number of institutional deliveries) was independently extracted from the electronic database by 2 different individuals and then compared. Cases of discordance were discussed and verified by returning to the HIS database (Développement Sanitaire du Mali) until consensus was reached among the data extractors. Access to data was facilitated by the fact that the authors carrying out this work were part of Mali's National Evaluation Platform, as Keita et al describe.^7^ This group of researchers includes members at the *Cellule de Planification et de la Statistique*, where HIS data are stored.

Table 1 compares HIS and DHS definitions of the 3 indicators, and Table 2 shows changes in indicator definitions in the routine HIS data over time.

**TABLE 1.** Indicators Definition According to Routine and Survey Data, Mali 2012

Indicators	HIS (Routine)		DHS	
	Numerator	Denominator	Numerator	Denominator
Contraceptive prevalence	Total number of women consulting family planning services in health facilities	Total number of women aged 15–49 years	Number of women aged 15–49 years at risk of getting pregnant that are using a method of contraception	Total number of women aged 15–49 years at risk of getting pregnant
Institutional delivery	Total number of births in community health facility and district hospital (public only)	Total number of births expected during the year	Number of children surveyed born in a health institution (private or public)	Total number of births
DPT3 vaccine	Number of children aged 0–11 months who received DPT3	Total number of children 0–11 months	Number of children aged 12–23 months who have received DPT3	Total number of children aged 12–23 months

Abbreviations: DHS, Demographic and Health Survey; DPT3, 3 doses of the diphtheria, pertussis, and tetanus vaccine; HIS, health information system.

**TABLE 2.** Routine Indicator Availability and Definition Change Over Time, 2001–2012, Mali

Indicators and Years of Availability	Years											
	2001	2002	2003	2004	2005	2006	2007	2008	2009	2010	2011	2012
Contraceptive prevalence rate, 2001–2012	Same definition over time											
Institutional delivery, 2007–2012	Same definition over time											
DPT3 vaccine, 2001–2012	DTCP3							DTCP3+HiB3		Penta3		

Abbreviations: DPT3, 3 doses of the diphtheria, pertussis, and tetanus vaccine; DTCP3, 3 doses of the diphtheria, tetanus pertussis, and poliomyelitis combined vaccine; HiB3, 3 doses of haemophilus influenzae type B vaccine; Penta3, 3 doses of pentavalent vaccine.

### Data Analysis

We calculated cluster-stratified survey-weighted coverage estimates and standard errors using DHS data for each indicator and survey at national and regional levels. To calculate estimates for routine data, we divided the numerator as reported in the HIS by the estimated population denominator (using population projections from the 2009 national census)^17^ at national and regional levels. We also calculated the standard errors for the survey and routine coverage estimates.

Because we had 3 survey estimates available, we defined 2 time intervals for comparison: 2001–2006 and 2006–2012. We visualized survey and routine coverage estimates with their 95% confidence intervals and compared the direction of the time trends in each interval.

To assess whether time trends from routine and survey data differed significantly from 2001 to 2006 or from 2006 to 2012, we standardized the difference of differences by subtracting the difference between 2001 and 2006 for survey data from the difference between 2001 and 2006 for routine data and dividing this quantity by the square root of the sum of survey and routine variance. Assuming a Gaussian distribution with mean 0, and standard deviation 1, we calculated the probability of a difference as or more extreme than the one we observed between survey and routine data. We reported *P* values for each comparison, aware that there is a 5% chance that a random observation from a Gaussian distribution will have a significant p-value, based on chance alone. We did not adjust *P* values for multiple comparisons.

Analyses were conducted using R version 3.5.1. All analysis files are publicly available: https://doi.org/10.5281/zenodo.5649508.

## RESULTS

### Data Availability

We were able to obtain coverage estimates from the routine reports and databases for the national level and all regions for the 3 indicators we examined from 2001 to 2012.

### National-Level Time Trends

**FIGURE 1:**
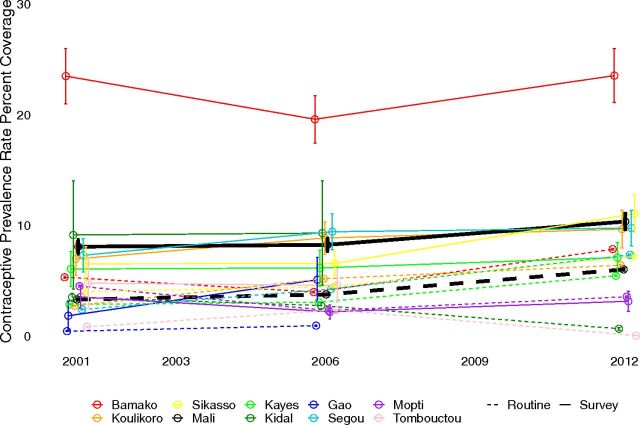
Contraceptive Prevalence Rate Time Trends by Survey and Routine Data, at National and Regional Levels From 2001 to 2012, Mali

**FIGURE 2:**
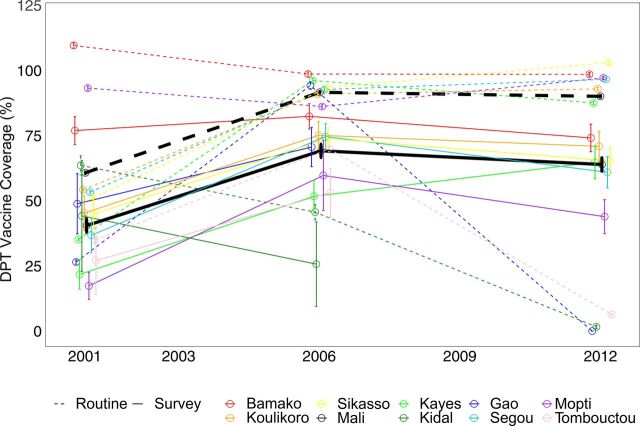
Diphtheria, Pertussis, and Tetanus Vaccine Coverage Time Trends at National and Regional Levels According to Routine and Survey Data From 2001 to 2012, Mali Abbreviation: DPT, diphtheria, pertussis, tetanus.

**FIGURE 3:**
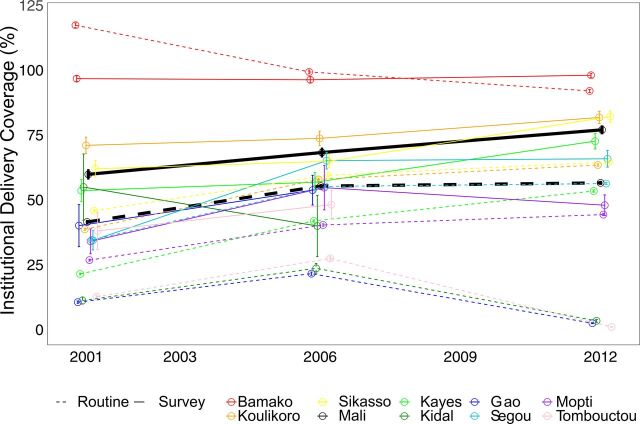
Institutional Delivery Rate Time Trends at National and Regional Levels According to Routine and Survey Data From 2001 to 2012, Mali

**FIGURE 4:**
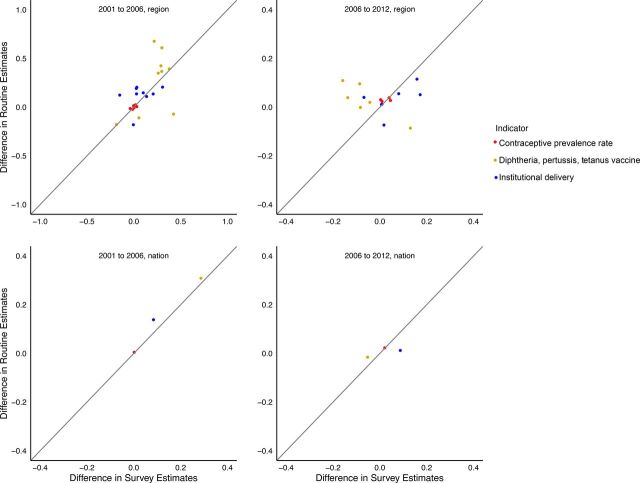
Prediction of Routine Annual Average Change for 3 Indicators by Survey Data, Comparing 2 Time Intervals and at Regional and National Levels, Mali^a^ ^a^Each dot represents the difference, from time 1 to time 2, in estimated proportion coverage, divided by the number of years in the time period.

Figures 1, 2, and 3 show time trends in CPR, DPT3, and institutional delivery, respectively, at national and regional levels using routine and survey data. Figure 4 shows the percentage point change for survey versus routine data. Based on survey data, time trends for CPR were essentially flat from 2001 to 2012, DPT3 increased sharply from 2001 to 2006 and declined slightly from 2006 to 2012, and institutional delivery increased slowly from 2001 to 2012. At the national level, the direction of the time trends was consistent between routine and survey data for all indicators and time periods. Notably, both routine and survey data identified a slightly negative trend in DPT3 coverage from 2006 to 2012. However, the magnitude of the time trends was significantly different between routine and survey data for institutional delivery, and, for 1 time period, for DPT3 (Tables 3 and 4). In addition, there were large differences between the point estimates from routine and survey data; routine data underestimated coverage of CPR and institutional delivery and overestimated DPT3 coverage relative to survey data.

**TABLE 3.** National Level Time Trend Change in Proportion Indicator Coverage According to DHS and Routine Data, 2001–2006, Mali

	Survey Data		Routine Data				
Indicator	2006 Estimate Minus 2001 Estimate	SE	2006 Minus 2001	SE	Difference Between Survey and Routine	Z Score	P Value
CPR	0.0017	0.0043	0.0045	0.0002	0.0028	−0.6505	.52
DPT3	0.2867	0.0183	0.3089	0.0018	0.0222	−1.2073	.23
ID	0.0848	0.0097	0.1384	0.0013	0.0536	−5.4768	0^a^

Abbreviations: CPR, contraceptive prevalence rate; DHS, Demographic and Health Survey; DPT3, 3 doses of diphtheria, pertussis, and tetanus vaccine; ID, institutional delivery; SE, standard error.

a Statistically significant.

**TABLE 4.** National Level Time Trend Change in Proportion Indicator Coverage According to DHS and Routine Data, 2006–2012, Mali

	Survey Data		Routine Data				
Indicator	2012 Estimate Minus 2006 Estimate	SE	2012 Minus 2006	SE	Difference Between Survey and Routine	Z Score	*P* Value
CPR	0.021	0.0046	0.0226	0.0002	0.0016	−0.3475	.73
DPT3	−0.0524	0.018	−0.0156	0.0018	0.0368	−2.0343	.04^a^
ID	0.0867	0.0087	0.0123	0.0013	0.0744	8.4578	0^a^

Abbreviations: CPR, contraceptive prevalence rate; DHS, Demographic and Health Survey; DPT3, 3 doses of diphtheria, pertussis, and tetanus vaccine; ID, institutional delivery; SE, standard error.

a Statistically significant.

### Regional-Level Time Trends

Time trends for all 3 indicators varied widely between regions, particularly for DPT3 and institutional delivery using routine data, and there was far less consistency between survey and routine time trends, relative to national estimates (Figures 1, 2, and 3). All regions had at least 1 statistically significant difference between routine and survey time trends except Tombouctou from 2001 to 2006 and Koulikoro from 2006 to 2012 (Tables 5 and 6).

**TABLE 5.** Regional Level Time Trend Change in Proportion Indicator Coverage According to DHS and Routine Data, 2001–2006, Mali

		Survey Data		Routine Data				
Region	Indicator	2006 Estimate Minus 2001 Estimate	SE	2006 Minus 2001	SE	Difference Between Survey and Routine	Z Score	*P* Value
Bamako	CPR	-0.039	0.0169	-0.0134	0.0006	0.0256	−1.5138	.13
Gao	CPR	0.0326	0.0122	0.0051	0.0004	0.0275	2.2529	.02^a^
Kayes	CPR	0.001	0.0116	0.002	0.0004	0.001	-0.0862	.93
Kidal	CPR	0.0017	0.0346	−0.0076	0.0024	0.0093	0.2681	.79
Koulikoro	CPR	0.0189	0.0107	0.0243	0.0004	0.0054	−0.5043	.61
Mopti	CPR	−0.0135	0.0063	−0.022	0.0004	0.0085	1.3465	.18
Ségou	CPR	0.0213	0.0113	0.0185	0.0004	0.0028	0.2476	.80
Sikasso	CPR	0.00041	0.0096	0.0045	0.0004	0.0044	−0.4579	.65
Tombouctou	CPR	−0.0021	0.0141	0.0153	0.00045	0.0174	−1.2333	.28
Bamako	DPT3	0.0546	0.037	−0.1098	0.0063	0.1644	4.3802	0^a^
Gao	DPT3	0.2176	0.0704	0.6773	0.0083	0.4597	−6.4849	0^a^
Kayes	DPT3	0.2998	0.0427	0.61	0.0045	0.3102	−7.2246	0^a^
Kidal	DPT3	−0.1849	0.1364	−0.1794	0.0239	0.0055	−0.0397	.97
Koulikoro	DPT3	0.2985	0.0433	0.367	0.0045	0.0685	−1.5735	.12
Mopti	DPT3	0.4235	0.0735	−0.0707	0.0053	0.4942	6.7064	0^a^
Ségou	DPT3	0.3788	0.0423	0.3933	0.0044	0.0145	−0.341	.73
Sikasso	DPT3	0.2901	0.037	0.4244	0.0041	0.1343	−3.6076	3,00E-04^a^
Tombouctou	DPT3	0.261	0.0812	0.3495	0.007	0.0885	−1.0859	.28
Bamako	ID	−0.0036	0.0078	−0.1801	0.0058	0.1765	18.1583	0^a^
Gao	ID	0.1381	0.0506	0.1098	0.0039	0.0283	0.5576	.58
Kayes	ID	0.0321	0.0289	0.2046	0.0028	0.1725	−5.941	0^a^
Kidal	ID	−0.15	0.0886	0.1242	0.0119	0.2742	−3.0673	.002^a^
Koulikoro	ID	0.0279	0.0215	0.1922	0.0033	0.1643	−7.5534	0^a^
Mopti	ID	0.2076	0.0502	0.1354	0.0029	0.0722	1.4359	.15
Ségou	ID	0.3082	0.0252	0.206	0.0031	0.1022	4.0252	1,00E-04^a^
Sikasso	ID	0.0293	0.0211	0.1365	0.0032	0.1072	−5.0231	0^a^
Tombouctou	ID	0.1021	0.0463	0.1477	0.0039	0.0456	−0.9814	.33

Abbreviations: CPR, contraceptive prevalence rate; DHS, Demographic and Health Survey; DPT3, 3 doses of diphtheria, pertussis, and tetanus vaccine; ID, institutional delivery; SE, standard error.

a Statistically significant.

**TABLE 6.** Regional Level Time Trend Change in Proportion Indicator Coverage According to DHS and Routine Data, 2006–2012, Mali

		Survey Data		Routine Data				
Region	Indicator	2012 Estimate Minus 2006 Estimate	SE	2012 Minus 2006	SE	Difference Between Survey and Routine	Z Score	*P* Value
Bamako	CPR	0.0395	0.0166	0.0388	0.00045	7,00E-04	0.0421	.97
Kayes	CPR	0.0094	0.0109	0.0239	0.0004	0.0145	−1.3294	.18
Koulikoro	CPR	0.008	0.0115	0.0124	0.0005	0.0044	−0.3822	.70
Mopti	CPR	0.0096	0.0056	0.0125	0.0004	0.0029	−0.5165	.61
Ségou	CPR	0.0033	0.0117	0.0308	0.0005	0.0275	−2.3483	.019^a^
Sikasso	CPR	0.0452	0.0102	0.0275	0.0004	0.0177	1.734	.08
Bamako	DPT3	−0.0828	0.0366	−0.0011	0.0054	0.0817	−2.2083	.027^a^
Kayes	DPT3	0.1304	0.045	−0.0859	0.0049	0.2163	4.7784	0^a^
Koulikoro	DPT3	−0.0425	0.0401	0.02	0.0045	0.0625	−1.5489	.12
Mopti	DPT3	−0.1581	0.0761	0.1096	0.0049	0.2677	−3.5105	4,00E-04^a^
Ségou	DPT3	−0.1369	0.0397	0.0392	0.0046	0.1761	−4.4063	0^a^
Sikasso	DPT3	−0.0859	0.0342	0.0959	0.0044	0.1818	−5.2723	0^a^
Bamako	ID	0.0166	0.007	−0.0733	0.0047	0.0899	10.6624	0^a^
Kayes	ID	0.1584	0.0248	0.1152	0.0031	0.0432	1.7285	.08
Koulikoro	ID	0.0795	0.0186	0.0554	0.0033	0.0241	1.2758	.20
Mopti	ID	−0.0685	0.0482	0.0402	0.003	0.1087	−2.2508	.024^a^
Ségou	ID	0.0059	0.0232	0.0111	0.0031	0.0052	−0.2222	.82
Sikasso	ID	0.1719	0.017	0.0513	0.0031	0.1206	6.979	0^a^

Abbreviations: CPR, contraceptive prevalence rate; DHS, Demographic and Health Survey; DPT3, 3 doses of diphtheria, pertussis, and tetanus vaccine; ID, institutional delivery; SE, standard error.

a Statistically significant.

CPR time trends at the regional level were not significantly different between routine and survey data; the only exceptions were Gao (2001–2006), and Ségou (2006–2012) (Tables 5 and 6). In addition, the direction of the CPR trends was consistent between routine and survey data except for Kidal and Tombouctou (2001–2006), although in both cases the difference between the routine and survey estimates was very small.

DPT3 trends were significantly different for 4 of 9 regions from 2001 to 2006 and 5 of 6 regions from 2006 to 2012 (Tables 5 and 6). In addition, the direction of the DPT3 time trend was different between routine and survey data for all 6 regions from 2006 to 2012. Similarly, institutional delivery time trends were significantly different between routine and survey data for 6 of 9 regions from 2001 to 2006 and 3 of 6 regions from 2006 to 12. The direction of time trends for institutional delivery was mostly consistent between routine and survey data, with 1 exception in 2001–2006 and 2 exceptions in 2006–2012.

## DISCUSSION

We aimed to compare routine and survey data trends over approximately 10 years at national and regional levels in Mali. We found that time trends for CPR, DPT3, and institutional delivery indicators in Mali were broadly similar between routine and survey data at the national level but were much more inconsistent at the regional level. This comparison is relevant to country and global stakeholders for several reasons. First, although household surveys are the preferred source for population-based measures of coverage, they are only available intermittently—every 3–5 years or even more infrequently— and therefore are of limited utility to support regular decision making.^18^ Second, routine data are available at low levels of disaggregation and would be a more granular alternative to survey data. Third, in Mali, researchers and planners already rely heavily on routine data.^3^ Given that the HIS is managed by Ministry of Health staff and that sense of country ownership over these data is high, routine data are more sustainable than externally coordinated and funded household surveys. However, it is important to understand to what extent these data may capture population changes in intervention coverage.

Previous studies have reported poor quality of routine data,^1^^,^^5^^,^^6^ but there have been limited assessments of external validity. Other analyses have found differences between routine and survey data with respect to point estimates,^18^ and some have found few identifiable patterns.^19^ We note that there were differences in indicator definition between survey and routine data (Table 5). This is frequently the case with survey and routine data because the data sources capture different kinds of data and may in part explain the differences we observed. However, routine data are often used to proxy survey data, so comparing the 2 data sources remains relevant. We focused primarily on the comparability of time trends rather than specific indicator levels, as both the direction and magnitude of time trends are often used by stakeholders to make decisions about which interventions or geographic areas to prioritize.

We focused primarily on the comparability of time trends rather than specific indicator levels, as both the direction and magnitude of time trends are often used by stakeholders to make decisions about which interventions or geographic areas to prioritize.

We found that national-level time trends were more comparable than regional-level trends, which may be due to the denominators used for the routine coverage estimates. Denominators for coverage indicators in routine data are typically based on projections from the most recent census. Internal migration—which would affect regional and district denominators but not national denominators—is often not captured in census projections. Depending on how recent these data are, the accuracy of the denominator may be affected.^20^ The most recent census available in Mali at the time of the analysis was held in 2009.^17^ An alternate approach for groups looking to replicate this analysis could be to use DHS-derived denominators for this analysis which would capture the distribution of women of reproductive age, births, and children by region.

The comparability of survey and routine data was generally better for CPR than for DPT3 and institutional delivery. This may be related to the fact that CPR changed very little from 2001 to 2012. In addition, the denominator for CPR, all women aged 15 to 49, is broader than the denominator for the other 2 indicators (pregnant women, and children aged 12–23 months) and may be less subject to error in census projections.^21^

We found that at the national level, routine data overestimated vaccine coverage but under-estimated CPR and institutional delivery. Women (and men) may access contraceptives outside of health facilities (e.g., private pharmacies), which may account for the underestimation of CPR. Similarly, women may give birth at private clinics, and these births are generally not captured in the routine HIS. Vaccination is generally delivered through the public health system, and overestimation of vaccination coverage in routine data is well-documented.^20^^,^^22^

Data extraction and cleaning for this analysis was a time- and labor-intensive process that required meticulous processing. Changes in district boundaries and indicator definition further complicated the process. Doing this rigorous, detail-oriented endeavor regularly is not realistically feasible. However, we note that at the time of analysis, Mali did not have the District Health Information System, version 2 (https://www.dhis2.org/) (DHIS2) in place.^23^ It is likely that the time and effort burden required for this process would have been considerably lighter if such a platform had already been established.^19^ With additional investments in building both more robust reporting systems^18^^,^^20^^,^^22^^,^^24^ and strong data use capacity including regular data quality assessments,^8^^,^^25^^,^^26^ routine data quality is likely to improve and this level of 1-time, in-depth data cleaning may not be necessary.

With the introduction of DHIS2 in Mali, more standardized indicator definitions will be used. The capacity of this platform to produce data visualizations at more granular levels (i.e., health facility or district level) and to increase detection of data quality issues at that level can lead to improved quality of aggregated data at the regional or national level. Building an information culture whereby managers are incentivized to use the data collected to make concrete changes in their health facility or district is a way to ensure that HIS data quality continues to improve.^27^ In a case study from Ethiopia, integrated supportive supervision— where managers aim to work with staff to review data and find solutions rather than adopting a punitive approach— has led to more accurate data being recorded and to data being used for decision making.^28^ While our findings currently do not support using routine data for impact evaluations, initiatives such as these could eventually result in data of sufficient quality to be appropriate for this purpose.

### Limitations

Because DHSs were conducted only every 5–6 years, we were not able to look at more granular time trends. Additionally, the 2012 DHS excluded 3 regions and several districts due to the security situation in these areas at the time of data collection. Because of this, we were unable to assess if our findings held true from 2006 to 2012 in excluded regions.

Furthermore, since household survey data are generally assumed to be of higher quality than routine data, we assumed that the DHS data represented “truth.” We recognize, however, that household survey data has its own set of data quality issues,^29^^,^^30^ and some household survey estimates may have substantial nonsampling error. In addition, regional estimates in the Mali DHS had wide confidence bounds due to relatively small sample sizes that may have limited our ability to detect significant differences between survey and routine data at the regional level.

We focused only on the external consistency of routine data and did not look at other data quality metrics, namely completeness, timeliness, internal consistency, and representativeness. Assessing these metrics may have led to a more complete picture of where data quality gaps exist and how they could be addressed. Taken together, these limitations may limit the validity of our findings if, for example, the DHS results did indeed have significant data quality issues or if other dimensions of data quality not explored in this article were of low quality.

## CONCLUSION

Given the frequent use of routine data in maternal, newborn, and child health programs in Mali, we aimed to assess the difference between indicator time trends from routine and household survey data to guide decision makers in Mali. Improving the data quality and accessibility of routine data is a high priority in many LMICs, and as part of this effort, it is important to assess the quality and usability of routine data in their current state. Trends in routine data appeared comparable to trends in household survey data at the national level and therefore may be appropriate for use at that level, but time trends in routine data should be interpreted with caution at the subnational level.

Given these findings, routine coverage data in Mali may not be suitable for impact evaluations, as evaluators need precise, accurate estimates of change to understand the extent to which a program is working. However, these data might be useful for planning and prioritization, if stakeholders keep in mind the potential error associated with subnational estimates. Given the potential for routine data to be a sustainable and timely source of appropriately disaggregated data, the push for improving the quality of routine data through exercises such as these should continue to be prioritized.

## Translation

**En Français**

**Peut-on utiliser les données de routine pour la prise de décision stratégique? Une comparaison des tendances temporelles entre les données d'enquête et de routine au Mali**

Contexte: Les pays aux ressources limitées ont besoin de données actualisées et de haute qualité sur la couverture des interventions de santé pour prendre des décisions stratégiques sur l'affectation des investissements dans la santé. Les données d'enquêtes auprès des ménages sont généralement considérées comme des données de références, de haute qualité. Cependant, les enquêtes sont coûteuses et menées infréquemment, alors que les décisions et les ajustements en matière de programmes et d'investissement doivent être prises beaucoup plus fréquemment. Cette étude a évalué la comparabilité des tendances temporelles de la couverture des interventions à partir des données de routine et d'enquête aux niveaux national et infranational au Mali.

Méthodes: Nous avons comparé 3 indicateurs de couverture: taux de prévalence contraceptive, accouchement médicalisé et 3 doses de vaccin contre la diphtérie, coqueluche et tétanos (DTC3), à l'aide de 3 Enquêtes Démographiques et de Santé du Mali (EDS 2001, 2006, et 2012–2013) et des données de routine du système de santé couvrant la période 2001–2012. Pour les données de routine, nous avons utilisé les rapports annuels du système local d'information sanitaire (SIS) et une base de données SIS pour extraire les données de chaque indicateur. Pour comparer les tendances temporelles entre les sources de données, nous avons calculé la variation en points de pourcentage et l'intervalle de confiance à 95% entre 2001–2006 et 2006–2012. Nous avons ensuite calculé les différences absolues et relatives entre les 2 sources de données pour chaque indicateur au fil du temps aux niveaux national et régional et évalué s'ils étaient statistiques significatifs.

Résultats: Nous avons constaté que la direction et l'ampleur des tendances temporelles du taux de prévalence contraceptive, de l'accouchement médicalisé et du vaccin DTC3 de 2001 à 2012 étaient similaires au niveau national entre les sources de données. Cependant, au niveau régional, il y avait des différences significatives dans l'ampleur et la direction des tendances temporelles pour l'accouchement en établissement et le vaccin DTC3, tandis que les tendances de la prévalence contraceptive étaient plus cohérentes. Les données de routine avaient tendance à surestimer la couverture du DTC3 et à sous-estimer l'accouchement médicalisé et la prévalence contraceptive par rapport aux données d'enquête.

Conclusion: Les données de routine au Mali – – en particulier au niveau national – – semblent appropriées pour être utilisées pour guider la planification et la hiérarchisation des programmes, mais les tendances temporelles de routine doivent être interprétées avec prudence au niveau sous-national. Cependant, pour les évaluations de programme, les données de routine peuvent ne pas être appropriées pour tirer des conclusions précises sur l'impact du programme.

**Peer Reviewed**

**First published online:** November 30, 2021.
